# Predicting ICU Admission Risk in Children with Respiratory Syncytial Virus

**DOI:** 10.1007/s40121-025-01155-w

**Published:** 2025-04-19

**Authors:** Young Hwa Lee, Young June Choe, Yoon Sun Yoon, Ji Young Park, Yun-Kyung Kim, Hyung Joon Joo, Sujin Choi, Hyun Jung Kim, Lorenzo Bertizzolo

**Affiliations:** 1 Allergy Immunology Center, Korea University, Seoul, Republic of Korea; 2 Department of Pediatrics, Korea University Anam Hospital, 73, Goryeodae-ro, Seongbuk-gu, Seoul, Republic of Korea; 3 Department of Pediatrics, Korea University College of Medicine, Seoul, Republic of Korea; 4 Department of Pediatrics, Korea University Guro Hospital, Seoul, Republic of Korea; 5 Department of Pediatrics, Korea University Ansan Hospital, Ansan, Republic of Korea; 6 Department of Cardiology, Cardiovascular Center, Korea University College of Medicine, Seoul, Republic of Korea; 7 Department of Medical Informatics, Korea University College of Medicine, Seoul, Republic of Korea; 8 Research Institute for Medical Bigdata Science, Korea University College of Medicine, Seoul, Republic of Korea; 9 Medical Affairs, Sanofi Pasteur, Seoul, Republic of Korea; 10 Medical Evidence Generation, Sanofi, Lyon, France

**Keywords:** Respiratory syncytial virus, RSV, Severe, Children, ICU

## Abstract

**Introduction:**

Respiratory syncytial virus (RSV) is a common infection in young children and a frequent cause of hospitalization. In some cases, RSV can lead to severe lower respiratory tract illness requiring admission to the intensive care unit (ICU). Here, we explore risk factors for RSV-related ICU admission in children.

**Methods:**

We conducted a retrospective study using Electronic Medical Record (EMR) data transformed into the Observational Medical Outcomes Partnership (OMOP) Common Data Model (CDM) from three tertiary care centers in Korea between 2008 and 2022. We identified 1529 children hospitalized with RSV according to the CDM and examined risk factors for ICU admission in this population.

**Results:**

Of 33,674 children aged 0–9 years who tested for RSV, 1529 (4.5%) were positive. The highest proportion of RSV-positive children were less than 10 months old. The ICU admission rate among RSV-positive children was 1.8% (29/1529), and the highest ICU admission rate occurred in children aged 0–5 months (4.4%). In a multivariable logistic regression model, we found that the odds of ICU admission were higher in younger age groups, with the highest odds of ICU admission occurring in children aged 0–5 months (aOR 10.39, 95% CI 2.33–46.29). We also found that gestational age less than 27 weeks was associated with a 71-fold increased odds of ICU admission (aOR 71.64, 95% CI 4.64–1106.50) and that extremely low birth weight was associated with a 31-fold increase in odds of ICU admission (aOR 31.16, 95% CI 2.35–414.00).

**Conclusions:**

We used the OMOP-CDM to identify risk factors for severe RSV infection requiring ICU admission in children. We found that young age, low gestational age, and low birth weight were associated with increased odds of ICU admission. Further research is needed to validate our findings and to examine other potential risk factors for severe RSV infection.

## Key Summary Points

**Table:** 

***Why carry out this study?***
Respiratory syncytial virus (RSV) is a common respiratory infection in children that can lead to serious complications, including hospitalization and admission to the intensive care unit (ICU).
Identifying risk factors for ICU admission among children with RSV infection is crucial for optimizing medical care and resource allocation.
***What was the hypothesis of the study? ***
This study aimed to identify risk factors associated with increased odds of ICU admission in children hospitalized with RSV infection using a standardized clinical information system.
***What was learned from the study?***
Young age (0–5 months), low gestational age, and low birth weight were identified as significant risk factors for ICU admission in children with RSV infection.

## Introduction

Respiratory syncytial virus (RSV) infection is a prevalent viral illness with a significant impact on the well-being of individuals, particularly young children and older adults. Over the past two decades, the incidence of RSV infection has substantially increased [1]. Lower respiratory tract infection (LRTI), the most severe complication of RSV infection, can lead to respiratory distress and an elevated risk of mortality [2]. Numerous studies have explored risk factors for severe RSV infection, including prematurity, low birth weight, crowded living conditions, chronic lung diseases, congenital heart diseases, and immunodeficiency [3]. Nevertheless, few studies have comprehensively investigated multiple risk factors concurrently [4]. Additionally, some studies have reported conflicting findings regarding specific risk factors [5].

Previous research on risk factors for severe RSV infection primarily relied on electronic medical records (EMR) or administrative claims databases. Although EMRs offer detailed clinical data, identifying a significant number of cases is time-consuming and laborious [6]. Furthermore, inconsistencies and variations in data formats across different EMR systems can complicate large-scale collaborative clinical research [7]. Observational studies using administrative claims databases can analyze large datasets across various regions or countries [8]. However, extracting and managing relevant clinical data for specific analyses can be challenging, as these databases are primarily designed for billing purposes.

Recent advances in medical informatics have enabled the development of standardized systems to unify diverse EMR data. In 2014, Observational Health Data Sciences and Informatics (OHDSI) introduced the Observational Medical Outcomes Partnership (OMOP) Common Data Model (CDM) to transform data—including patient demographics, diagnoses, and laboratory results—into a uniform format for streamlined analysis [9]. While previous studies have validated the reliability of the CDM, its application to respiratory infections in children is novel [10]. Our study leverages the OMOP-CDM to analyze a 14-year multicenter dataset from three Korean tertiary centers, confirming known risk factors such as low gestational age and low birth weight, and demonstrating the added value of this approach in pediatric respiratory research.

We aimed to provide deeper insight into the association between RSV infection and ICU admission in children. We have leveraged the real-world OMOP CDM database, offering access to relevant data in a large population-based study.

## Methods

### Study Design and Data Source

We conducted a retrospective cohort study using the OHDSI OMOP-CDM at Korea University Medical Center. The data comprised three tertiary referral centers in the Seoul-Gyeonggi metropolitan area: Korea University Anam Hospital, Korea University Guro Hospital, and Korea University Ansan Hospital. These hospitals collectively provide 2949 beds and serve approximately 830,000 hospitalized patients annually.

The Korea University Anam Hospital IRB Committee (IRB No. 2022AN0400) approved the study protocol and waived the requirements for informed consent.

The OMOP CDM is a structured data model using a standardized vocabulary. All medical and cost data were extracted, transformed, and loaded into OMOP CDM version 5.3 following OHDSI protocols. This process included diagnoses, drug exposures, and comprehensive medical practice records. Stringent data verification procedures were implemented using the Automated Characterization of Health Information at Large-Scale Longitudinal Evidence Systems, complemented by thorough checks performed by both data analysts and clinicians.

The EMR data from the three tertiary centers were transformed into the OMOP CDM using a standardized process. This transformation enabled us to capture a wide range of clinical variables, including patient demographics, gestational age, birth weight, palivizumab prophylaxis, and comorbid conditions. We also conducted a detailed assessment of data completeness and addressed missing values—for example, missing gestational age records were noted in a small subset of patients and appropriately handled during the analysis

We searched the CDM database for cases of primary RSV infections. Subsequently, using concept IDs for RSV infections, we identified cases of health outcomes recorded in the CDM database. The primary outcome under consideration was admission to the ICU during hospitalization.

### Study Population

We conducted an analysis using the OMOP-CDM database, which comprised data from 33,674 children admitted to the Korea University Medical Center between August 2008 and December 2022.

The eligibility criteria for RSV testing in hospitalized children were defined based on the following parameters: (1) age between 0 and 9 years; (2) hospitalization for a minimum of 1 day; and (3) undergoing RSV testing through multiplex polymerase chain reaction (PCR). Children were eligible for inclusion in the study only if they underwent RSV testing by multiplex PCR. Only those cases with PCR-confirmed RSV infection were included in the analysis, ensuring high diagnostic accuracy. Antigen testing was not performed and therefore not considered in our inclusion criteria, minimizing variability in the diagnostic approach and reinforcing the reliability of our cohort selection. Subsequently, the RSV-positive group was categorized as follows: (1) children admitted to the ICU (either neonatal ICU or pediatric ICU) during hospitalization, referred to as the ICU group; and (2) children who were not admitted to the ICU throughout hospitalization, referred to as the non-ICU group.

### Variables of Interest

Following a comprehensive literature review to identify potential risk factors for RSV infection leading to ICU admission, our analysis incorporated specific clinical data extracted from the CDM database. This data included demographic characteristics (age and sex), pre-existing medical conditions, gestational age, birth weight, and palivizumab prophylaxis, all collected during the RSV infection period. However, certain variables were excluded due to their absence in the electronic medical record (EMR) or inability to be translated into the CDM format. These excluded variables were as follows: length of observation, socioeconomic factors, presence of siblings, and maternal age.

### Statistical Analysis

Statistical analyses were performed using SAS software (version 9.4; SAS Institute, Cary, NC, USA). Descriptive statistics are presented as mean ± standard deviation (SD), median, counts, and percentages, as appropriate for the data type. Categorical variables were assessed using the chi-squared test, with RSV infection or ICU admission as the dependent variable and other variables as independent factors. Continuous variables were analyzed using a *t* test.

To identify risk factors associated with RSV infection and ICU admission, we employed a multivariate logistic regression model to compute odds ratios (ORs) and corresponding 95% confidence intervals (CIs). Statistical significance was set at *p* < 0.05. In the case of multicollinearity between gestational age and birth weight, two distinct regression analyses were conducted, with each analysis incorporating either gestational age or birth weight as an independent variable.

## Results

**Fig. 1:**
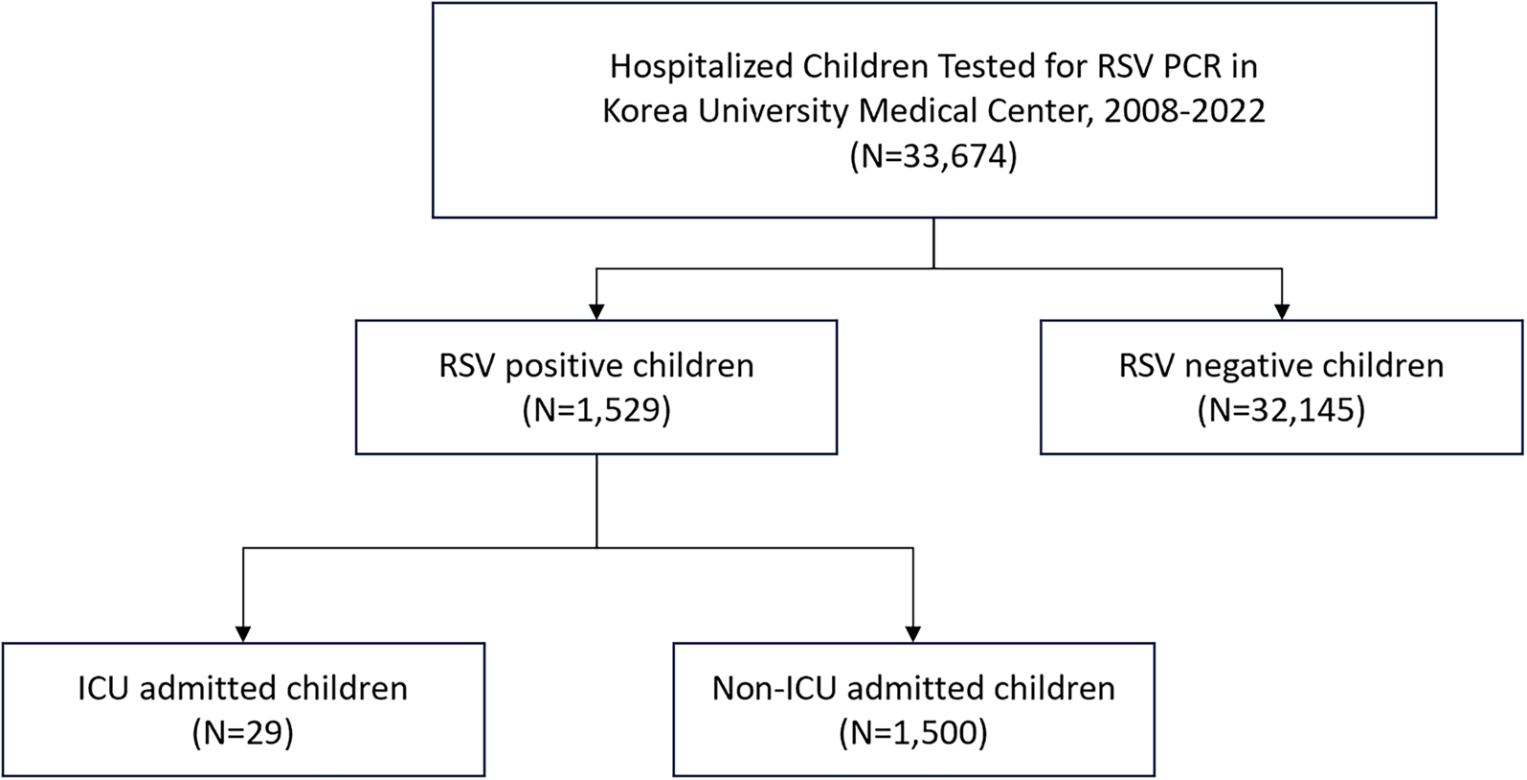
Selection process for hospitalized children with respiratory syncytial virus (RSV) and intensive care unit (ICU) or non-ICU admissions in Korea, from 2008 to 2022

Among 33,674 children tested for RSV by PCR at Korea University Medical Center, 1529 (4.5%) were positive (Fig. 1). RSV-positive children were approximately 10 months younger than those who tested negative. Children over 60 months of age had a significantly lower RSV infection rate (0.7%) compared with their RSV-negative counterparts. The proportion of children with and without a history of palivizumab prophylaxis was similar in both RSV-positive and RSV-negative groups. Other risk factors, such as gestational age, birth weight, and congenital heart disease were more prevalent among RSV-positive children (Table 1).

**Table 1:** Overview of demographic characteristics of hospitalized children tested for respiratory syncytial virus (RSV) in Korea from 2008 to 2022

	All RSV tested children *n* (%) (*n *= 33,674)		RSV negative children *n* (%) (*n *= 32,145)		RSV positive children *n* (%) (*n *= 1529)		*p* value
Age at diagnosis (month)							
Mean ± SD [median]	26.26 ± 27.09 [17.0]		26.71 ± 27.42 [17.0]		16.70 ± 16.29 [12.0]		
Year of hospitalization							< 0.0001
2008ￚ2012	6335	18.8	6163	19.2	172	11.2	
2013ￚ2017	17,793	52.8	17,446	54.3	347	22.7	
2018ￚ2022	9546	28.4	8536	26.5	1010	66.1	
Sex							0.5599
Male	18,873	56.1	18,005	56.0	868	56.8	
Female	14,801	43.9	14,140	44.0	661	43.2	
Age							< 0.0001
0ￚ5 months	8817	26.2	8337	25.9	480	31.4	
6ￚ11 months	4328	12.9	4076	12.7	252	16.5	
12ￚ23 months	7308	21.7	6922	21.5	386	25.3	
24ￚ59 months	8633	25.6	8255	25.7	378	24.7	
5-9 years	4588	13.6	4555	14.2	33	2.2	
Palivizumab prophylaxis							0.8249
Yes	121	0.4	115	0.4	6	0.4	
No	33,553	99.6	32,030	99.6	1523	99.6	
Gestational age^a^							< 0.0001
≤27 weeks	26	0.1	21	0.1	5	0.3	
28-36 weeks	1105	3.3	1020	3.2	85	5.6	
≥37 weeks	32,483	96.6	31,044	96.7	1439	94.1	
Birth weight							< 0.0001
ELBW	101	0.3	92	0.3	9	0.6	
LBW	576	1.7	529	1.6	47	3.1	
Normal	32,993	98.0	31,520	98.1	1473	96.3	
Congenital heart disease							< 0.0001
Yes	1878	5.6	1745	5.4	133	8.7	
No	31,796	94.4	30,400	94.6	1396	91.3	

*ELBW* extremely low birth weight (< 1000 g), *ICU* intensive care unit (including both neonatal and pediatric ICU), *LBW* low birth weight (1000–2500 g), *SD* standard deviation

^a^Excluding missing records of 60 patients

Of the 1529 RSV-positive children, 1500 did not require ICU admission, while 29 children did (Table 2). Children admitted to the ICU were approximately 10 months younger than those who were not admitted. Among all age groups, RSV-positive children aged 0–5 months had the highest ICU admission rate (4.4%). Children at a higher risk of ICU admission, including those with gestational age < 27 weeks (20.0%), extremely low birth weight (11.1%), and congenital cardiac disease (3.8%), displayed significantly higher ICU admission rates than those without ICU admission. Odds ratios were also significantly higher for children with gestational ages of 28–36 weeks and LBW.

**Table 2:** Comparative distribution of potential risk factors in respiratory syncytial virus (RSV)-positive children between ICU and non-ICU admissions in Korea from 2008 to 2022

	All RSV-positive children, *n* (%)		Non-ICU admitted children, *n* (%)		ICU admitted children, *n* (%)		*p* value
	(*n *= 1529)		(*n *= 1500)		(*n *= 29)		
Age at diagnosis (month)							
Mean ± SD [median]	16.70 ± 16.29 [12.0]		16.89 ± 16.33 [13.0]		7.03 ± 10.82 [3.0]		
Sex							0.5798
Male	868	56.8	853	56.9	15	51.7	
Female	661	43.2	647	43.1	14	48.3	
Age							< 0.0001
0ￚ5 months	480	31.4	459	30.6	21	72.4	
6ￚ11 months	252	16.5	249	16.6	3	10.3	
12ￚ23 months	386	25.2	383	25.5	3	10.3	
24ￚ59 months	378	24.7	376	25.1	2	7.0	
5–9 years	33	2.2	33	2.2	0	0.0	
Palivizumab prophylaxis							0.0079
Yes	6	0.4	5	0.3	1	3.5	
No	1523	99.6	1495	99.7	28	96.5	
Gestational age							0.0002
≤27 weeks	5	0.3	4	0.3	1	3.5	
28–36 weeks	85	5.6	80	5.3	5	17.2	
≥37 weeks	1439	94.1	1416	94.4	23	79.3	
Birth weight							0.0004
ELBW	9	0.6	8	0.5	1	3.4	
LBW	47	3.1	43	2.9	4	13.8	
Normal	1473	96.3	1449	96.6	24	82.8	
Congenital heart disease							0.0993
Yes	133	8.7	128	8.5	5	17.2	
No	1,396	91.3	1,372	91.5	24	82.8	
Death							< 0.0001
Yes	2	0.1	0	0.0	2	6.9	
No	1527	99.9	1500	100.0	27	93.1	

*ELBW* extremely low birth weight (< 1000 g), *ICU* intensive care unit (including both neonatal and pediatric ICU), *LBW* low birth weight (1000–2500 g), *SD* standard deviation

We employed two distinct logistic regression models to determine odds ratios for ICU admission to address the issue of multicollinearity between gestational age and birth weight (Table 3). Compared to the 24–59 month age group, the odds ratio for ICU admission varied across younger age groups, ranging from 1.50 (95% CI 0.25–9.15, *p =* 0.6626) in the 24–59 month category to 10.39 (95% CI 2.33–46.29, *p =* 0.0021) in the 0–5 month category. Variables such as sex and congenital cardiac disease were not statistically significant. Children with a gestational age < 27 weeks exhibited an odds ratio of 71.64 (95% CI 4.64–1106.50, *p = *0.0022) compared to those with a gestational age of 37 weeks and above. Similarly, the odds ratio for individuals with extremely low birth weight was 31.16 (95% CI 2.35–414.00, *p =* 0.0092) compared to those with normal birth weight.

**Table 3:** Multivariate analysis of potential risk factors for ICU admission in respiratory syncytial virus (RSV)-positive children in Korea from 2008 to 2022

Variables	Odds ratios (95% CI)	*p* value^a^
Sex		
Male	Reference	
Female	1.36 (0.64–2.90)	0.4200
Age		
5–9 years	–	
24–59 months	Reference	
12–23 months	1.50 (0.25–9.15)	0.6626
6–11 months	2.11 (0.34–13.05)	0.4202
0–5 months	10.39 (2.33–46.29)	0.0021
Congenital heart disease		
No	Reference	
Yes	0.61 (0.17–2.24)	0.4564
Gestational age^a^		
≥37 weeks	Reference	
28–36 weeks	5.04 (1.59–15.99)	0.0061
≤27 weeks	71.64 (4.64–1106.50)	0.0022
Birth weight^b^		
Normal	Reference	
LBW	7.35 (1.95–27.77)	0.0033
ELBW	31.16 (2.35–414.00)	0.0092

^a^*p* values except for sex and congenital cardiac disease were under 0.05

^b^Model 1 adjusted for gestational age; model 2 adjusted for birth weight

## Discussion

We aimed to determine the occurrence of severe RSV infection necessitating ICU admission in children below 10 years of age and to investigate associated risk factors for ICU admission using the CDM. We assembled a large cohort of 1529 pediatric patients hospitalized with RSV infection identified through the CDM database, providing a rich dataset for analyzing clinical outcomes. Our findings revealed that young age (0–5 months), low gestational age, and low birth weight were all associated with a high risk of ICU admission in children. Significantly, to our knowledge, this is the first study to utilize the CDM for clinical research specifically targeting childhood respiratory infections. While previous CDM applications have primarily focused on pharmaco-epidemiologic investigations related to disease treatment and epidemiological analyses of disease-related mortality across diverse research domains [11, 12], our study focused on identifying clinical risk factors for RSV infection leading to ICU admission in children, including comorbidities. This novel approach successfully revealed these risk factors.

Previous reports, often based on national registries or claims databases, have documented incidence rates of severe RSV infections of approximately 20–30% in Korea [13]. However, studies conducted within single institutions have generally reported lower incidences, typically ranging approximately 7–10%. This variation has been attributed to stringent definitions of RSV in single-institution studies. Our investigation, identified a lower proportion of RSV-related ICU admissions in children aged 0–9 years of (1.8%), with a higher rate of 4.4% among infants aged 0–5 months, which is lower than previously reported ICU admission rates of 15–19% [14]. Further multicenter studies with larger cohorts are essential to determine if these variations represent institutional or regional disparities in severe RSV outcomes. Our findings regarding the association between age and severe RSV infection risk align with previous research [15]. Specifically, our results support existing evidence that preterm infants or those with congenital heart disease may have compromised immune function, leading to a higher risk of infection [16, 17].

This study has several limitations. First, the identification of RSV cases relied on the assumption that all patients with RSV-LRTIs underwent RT-PCR testing. However, some patients with mild RSV infections may not have been tested, potentially underestimating the RSV cases. Furthermore, our study population was restricted to children who received medical care and testing within a single healthcare system. Excluding individuals hospitalized elsewhere following RSV infection management at the Korea University Medical Center may also contribute to underestimating the RSV burden. Second, despite including previously established risk factors, certain variables were excluded due to their absence in the EMR and inability to be converted into the CDM. In our univariate analysis, congenital cardiac disease was observed more frequently among ICU-admitted patients compared to non-ICU patients. However, in the multivariable logistic regression analysis—which adjusted for potential confounders such as age, gestational age, and birth weight—the association between congenital cardiac disease and ICU admission was attenuated and did not reach statistical significance (aOR 0.61, 95% CI 0.17–2.24, *p = *0.4564). This finding suggests that, while congenital cardiac disease may appear more common in the ICU group in unadjusted comparisons, its impact on ICU admission risk is likely overshadowed by stronger determinants such as low gestational age and low birth weight. Finally, although we established a substantial cohort of 1529 patients with RSV infection, our analysis relied solely on data from this particular healthcare system, potentially limiting its generalizability to broader populations. Another limitation is the small number of ICU admissions (*n* = 29), which resulted in wide confidence intervals and limited the precision of our risk estimates.

## Conclusion

Our study leveraged the OMOP-CDM to quantify the burden of RSV infection in Korean children and to identify key risk factors for ICU admission, notably low gestational age and low birth weight. These findings underscore the value of early risk assessment in identifying high-risk patients, which may inform clinical decision-making and guide future interventional research. Moreover, our work demonstrates the utility of the CDM in investigating pediatric clinical risk factors and lays the groundwork for further research in this area.

## Data Availability

Materials described in the manuscript, including relevant raw data, will be made freely available to researchers for non-commercial purposes, while ensuring the confidentiality of the participants. Analytical SAS code was obtained from choey@korea.ac. kr.
